# Can We Minimize the Risk of Dogs Developing Canine Otitis Externa?—A Retrospective Study on 321 Dogs

**DOI:** 10.3390/ani14172537

**Published:** 2024-08-31

**Authors:** Peter Christian Ponn, Andrea Tipold, Andrea Vanessa Volk

**Affiliations:** 1 AniCura Recklinghausen—Small Animal Clinic, 45659 Recklinghausen, Germany; 2 Department of Small Animal Medicine and Surgery, University of Veterinary Medicine Hannover, 30559 Hannover, Germany; andrea.tipold@tiho-hannover.de (A.T.); andrea.volk@tiho-hannover.de (A.V.V.)

**Keywords:** dermatology, canine, otitis externa, obesity, retrospective, prophylaxis

## Abstract

**Simple Summary:**

In daily veterinary practice, canine otitis externa is a common presentation and, therefore, many studies were conducted to evaluate predispositions. This study on the other hand aims to evaluate factors that have potentially statistically reduced risks for developing otitis externa. Regarding the results, Rhodesian Ridgebacks and Collies have shown significantly low odds for otitis externa. Furthermore, regarding dogs with endocrinopathies, it was shown that those who are overweight seem to develop otitis externa more likely than those with a normal weight. Especially the latter result should be used in communication with pet owners to point out the importance of strict weight management and, therefore, increasing the quality of life of their dogs.

**Abstract:**

**Background:** Canine otitis externa is a common presentation in small animal practice. The clinical signs vary individually from patient to patient. Regardless of the expression, they all decrease the quality of life. Therefore, this study aimed to identify factors that showed reduced odds for otitis externa. **Methods:** Clinical case records were searched for key terms regarding canine otitis. Statistical analyses were applied to evaluate associations with otitis externa. *p*-values of <0.05 were considered statistically significant. **Results:** Rhodesian Ridgeback and dogs with underlying infectious diseases had a reduced risk for otitis externa, while Retrievers and dogs with allergies had an increased risk. Furthermore, in the group of dogs with endocrinopathies, those with normal weight showed a reduced risk of developing canine otitis in comparison to those which were overweight. **Conclusions:** These results show a new point of view to reduce the prevalence of canine otitis by reducing the animals’ weight due to an additional risk of otitis in obese or overweight dogs. Furthermore, for the first time, Rhodesian Ridgebacks were identified in our study population to have a lower risk of developing otitis externa. Thus, breeders should reflect—within other responsibilities for the quality of life in their offspring—on breed-specifics about otitis when planning ahead.

## 1. Introduction

Dogs with ear diseases make up a substantial part of patients in daily veterinary practices. Depending on the manifestation of inflammation, the condition is divided into otitis externa (the external ear canal including the pinna), otitis media (middle ear) and otitis interna (inner ear) [1,2,3,4,5].

In particular, otitis externa (OE) is one of the most common diseases in dogs with prevalence ranging from 4.58% [6] to 7.30% in general practice [7]. Some studies even estimate a prevalence of 10 to 20% of the dog population [8,9], a substantial number of canine patients suffering. The suffering entails pruritus, pain, impaired to loss of hearing and debilitating clinical signs, which are reducing quality of life moderately to severely [10]. To help these patients, knowledge and a structured work-up of possible etiologies of canine OE are important for treatment success. The etiologies have been classified in the PSPP system: primary, secondary, predisposing and perpetuating factors [10,11].

Predisposing factors in particular do not cause OE itself but increase the risk of developing an inflammation of the external ear canal, e.g., moisture, pendulous ears or ear canal conformation. Predisposing factors tend to influence the microenvironment and microflora, altering the microclimate and facilitating bacterial infections [4,12,13,14]. Several of the predisposing factors are breed-specific, like pinnal and/or ear canal formation, or behavior-related (hunting/swimming).

Primary and secondary factors should be viewed as a whole, since primary factors (hypersensitivities, ectoparasites, foreign bodies, etc.) cause per se inflammation, damage the epithelial layer in the ear canal and lead to an excessive increase in infectious agents and the secondary further enhancement of the inflammatory process [15,16,17].

Primary factors for OE include allergic dermatitis [7,15,18,19], with the most common being environmental allergies, flea bite allergies and cutaneous adverse food reactions, but also foreign bodies, immune-mediated diseases, endocrinopathies and keratinization disorders (e.g., in Cocker Spaniels) [6,7,15].

Furthermore, many diseases can manipulate the skin barrier and cause inflammation of the skin itself. As the ear canal is an appendix to the skin, this includes OE, concluding that in many cases, OE is most commonly secondary to an underlying disease [7,10,20,21].

Perpetuating factors are often the reason for treatment failure as they maintain the inflammatory process and can be found in many cases of chronic OE [11,22].

The treatment of OE includes managing the secondary infections first with concurrent pain management and cleaning practice by the owner. Important emphasis has to be put on topical management strategies for the individual owner, as topical therapy is a key factor in treating otitis. This is due to the fact that therapeutic concentrations are unlikely to be achieved by systemic antibiotics [18,23]. Depending on the duration of the disease and the severity and frequency of recurrence, primary and perpetuating factors should be addressed. The diagnostics necessary are often time- and cost-intensive in concurrence with difficult and time-intensive treatment applications by the owner [18]. In some cases, aural lavage [24,25] under general anesthesia becomes necessary, increasing costs even further. In cases, where particularly medical therapy is not possible or initiated too late to change the disease progression, the surgical removal of the affected ear canal(s), a salvage procedure, is needed [26]. This procedure is highly painful, costly and a life-long hearing impairment for the patient is common [26].

Therefore, a different approach is needed for our dogs to prevent the occurrence or even minimize the risk of OE. While most studies focus on potential predispositions and factors that increase the risk of OE, this study aims to determine features and phenotypic expressions which have a statistically low risk of OE.

## 2. Materials and Methods

### 2.1. Study Population

The cases selected for this study were chosen from our patient–management system (EasyVet^®^, VetZ GmbH, Isernhagen, Germany) based on the history ‘there is something wrong with my dog’s ear’. Additionally, cases presented in the dermatology unit, which had any dermatological disease besides OE and were registered with an updated medical history questionnaire newly established in 2020, were included. This way, the case population included a randomized control group, which, even though they had a dermatological problem, did not develop OE. This resulted in a total study population of 321 dogs.

**Figure 1:**
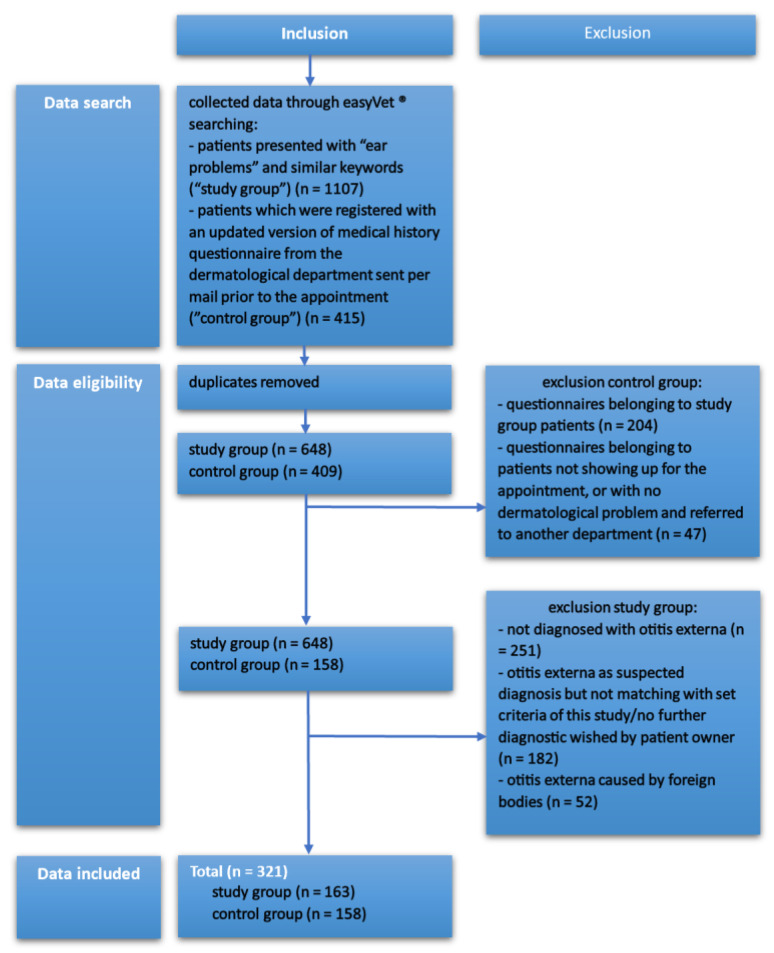
A run-down on the inclusion and exclusion of initial data, leading to a total number of 321 cases included in this study.

The data were collected between 1 January 2020 and 31 December 2021 in the small animal clinic (University of Veterinary Medicine Hannover, Hannover, Germany). Dogs with foreign bodies in their external ear canal were excluded from this study as these had a lesser connection to the phenotypic expression due to the fact that these cases were more closely related to their environment and lifestyle, which could have falsified the results. Patients which were presented multiple times in this period were counted as one subject. Figure 1 shows the summarized explanation of the selection process.

Patients suffering from OE were included if they showed at least two of the following signs: clinical features (e.g., inflammation, hyperemia, ulceration of the ear canal with pruritus, or stenosis ceruminous discharges) and/or cytological abnormalities (e.g., inflammatory cells or an increased amount of bacteria/yeast) [7,16,18].

### 2.2. Signalement

All dogs were grouped according to the following factors: the presence or absence of OE, breed, sex, neutering status, bodyweight, age and the underlying disease.

The breeds were classified as numbers to represent the referring breed (e.g., 1 = “Beagle”; 2 = “Boxer”; etc.). Breeds that were represented four times or less were classified in groups from A to D. This means breeds that were represented one time were put together in “Group A”; “Group B” summarized breeds that had two study subjects in the dataset, as in previous studies [6]. These categories were chosen to exclude these groups from further breed-related analysis to reduce the risk of falsification.

Group A (36 breeds): Sloughi, Puli, Cane Corso Italiano, German Longhaired Pointer, Cairn Terrier, Hovawart, Afghane Hound, Shetland Sheepdog, Bloodhound, Landseer, Caucasian Shepherd Dog, Podenco, Stabyhoun, Newfoundland, Terrier, Standard Schnauzer, German Jagd Terrier, Portuguese Sheepdog, English Springer Spaniel, German Wirehaired Pointer, Alaskan Malamute, Great Pyrenees, Australian Kelpie, Nova Scotia Duck Tolling Retriever, Cavalier King Charles Spaniel, Beauceron, Malteser, Spanish Greyhound, Shi Tzu, Bichon Frise, Chinese Shar-Pei, Basset Hound, Grand Basset griffon vendéen, German Shorthaired Pointer and Jagdterrier.

Group B (13 breeds): Bernese Mountain Dog, Staffordshire Bullterrier, Elo, Hanoverian Scenthound, Siberian Husky, Japanese Akita-Inu, Doberman Pinscher, Appenzell Mountain Dog, Gordon Setter, West Highland White Terrier, Weimaraner, Wolf and Irish Wolfhound.

Group C (11 breeds): American Bulldog, Miniature Bullterrier, Dachshund, Havanese, Small Munsterlander, Pomeranian, Australian Shepherd, American Pit Bull Terrier, Bordon Terrier, Parson Russell Terrier and Yorkshire Terrier.

Group D (2 breeds): Jack Russell Terrier and Magyar Vizsla.

Crossbreeds were counted as a group by themselves.

Each breed with more than four occurrences was analyzed independently.

In the case of bodyweight, on the one hand, it was analyzed metrically in kg. On the other hand, subjects were assigned to groups according to their difference to the normal weight range of the breed. As opposed to the hospital, where the body condition was rather documented in total kg than the body condition score, a different approach of classification was performed, leading to following seven weight classes. These classes are similar to body condition scoring [27]: cachectic (more than 30% difference to the lower limit), underweight (11–30% difference to the lower limit), slightly underweight (1–10% difference to the lower limit), normal, slightly overweight (1–10% difference to the upper limit), overweight (11–30% difference to the upper limit) and obese (more than 30% difference to the upper limit). The data of the American Kennel Club^®^ (American Kennel Club, Raleigh, NC, USA) were used for each breed to determine the normal bodyweight [28]. If the American Kennel Club^®^ (AKC) did not acknowledge the breed or the data did not differentiate between male and female weights, entries from the United Kennel Club^®^ (United Kennel Club; Kalamazoo; Michigan; USA) were used [29]. Dogs under the age of one were not included in the calculation of bodyweight as their normal weight differed from the standard range. The intention of this approach was to examine a correlation between the occurrence of OE and the body frame or over- and underweight. Crossbreeds were not included in this analysis.

Age was analyzed in the same ways: metrically and according to age groups. Metrically, the age was counted in months. Furthermore, the study population was again divided into five, this time with age groups, with the following structure: puppy (<1 year), young dog (1–3 years), middle-aged dog (4–6 years), old dog (7–10 years) and geriatric dog (>10 years). The age groups are based on the thresholds described by Naomi D. Harvey, but were adjusted to show an even distribution [30].

As OE in many cases is associated with an underlying disease, the latter (if diagnosed) was divided into the groups inflammatory, cryptogenic, allergic, endocrine, infectious, tumorous and other. Patients with a sole diagnosis of OE without further diagnostics at our hospital were classified as “inflammatory underlying disease”. However, both “inflammatory” and “other” underlying diseases were not used for further analysis to prevent falsifying results and so are not presented in Figure 4. Cryptogenics were used for cases that were nonresponsive to treatment or reoccurred with a further diagnostic approach not showing any hints of an underlying disease, as described in other studies [16]. For the infectious diseases, each case caused by parasites (e.g., *Ctenocephalides* spp., *Demodex* spp., etc.), bacterial and fungal diseases, but also diseases caused by infectious protozoals (e.g., *Leishmania* spp., *Anaplasma* spp.) was counted. Viral infections would have been counted as well, but did not occur in this dataset.

### 2.3. Clinical Examination

Complete physical and dermatological examinations including otoscopy were performed in all the cases by the vet on duty. Most of the dogs were presented to the dermatology department during out of hours’ duty. In every case with a potential OE, cytological samples were collected and processed in the widely practiced way (rolled out onto glass slides, air dried, stained with Romanowski stain (RAL Diff-Quick^®^; CellaVision-RAL Diagnostics, Lund, Sweden)) and examined (semiquantitative evaluation of identified microorganisms and inflammatory cells) [31,32].

### 2.4. Statistical Analysis

For datasets with metric variables, both the Shapiro–Wilk and the Kolmogoroff–Smirnov test were used to test for normal distribution. These tests were not applied to categorical datasets as nominal data do not come with a normal distribution. Subsequently, the Eta coefficient test was applied to the metric data to determine the strength of the association between the data and the presence of OE.

The Chi²- and Fisher’s exact test were used to assess the associations between two categorical variables. *p*-values < 0.05 were considered statistically significant. Furthermore, results that were statistically significant underwent Post Hoc Testing using the Bonferroni correction to minimize the risk of a type I error.

In the cases of the datasets of the sex, neutering status, age (categorized), weight (categorized) and some combined datasets, an odds ratio test was performed.

These combined datasets included: young dogs with a normal weight compared with old overweight dogs, dogs with a normal weight and an endocrinopathy compared with overweight dogs with an endocrinopathy, male-neutered individuals compared with female-intact and, lastly, male-intact compared with female-neutered subjects. The datasets including weight were chosen to further evaluate the potential influence of obesity. The gender-related datasets that were chosen as prior studies showed different results regarding the influence of gender [6,7,15,33].

Analyses were carried out using the IBM SPSS Statistics 27.0.1.0™ (IBM, Armonk, NY, USA) software.

## 3. Results

### 3.1. Study Population

**Figure 2:**
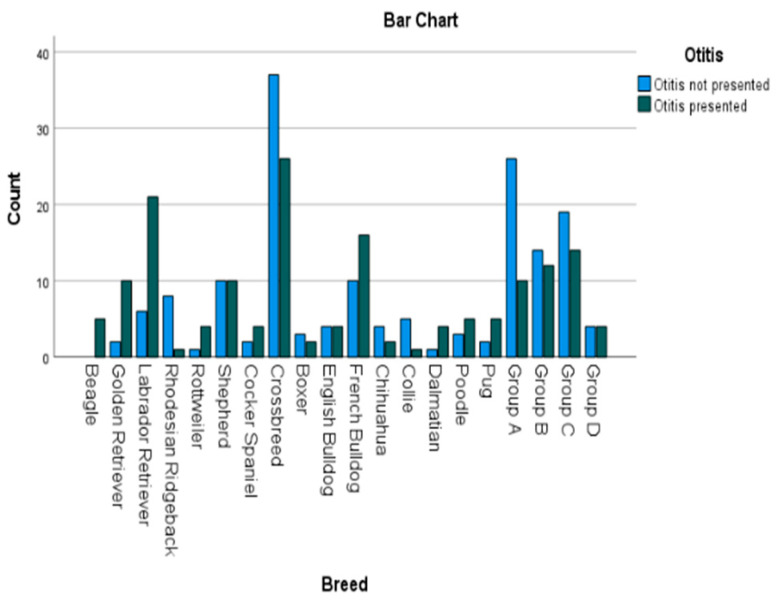
Bar charts displaying different kinds of breeds divided by their presence of OE. The breeds are shown on the *x*-axis and the number of cases on the *y*-axis. German Shepherds, Belgian Shepherds and White Swiss Shepherds were counted as “Shepherd” and Border Collies and Rough Collies as “Collie”.

Out of the 321 cases, 163 (50.7%) were diagnosed with OE; those cases are seen in Figure 2, grouped by breeds. There were 133 (41.4%) female and 188 (58.6%) male subjects, 148 (46.1%) dogs were neutered and 173 (53.9%) dogs were intact. Out of the female subject group, 61 (45.9%) presented with OE and 72 (54.1%) did not. In the male study population, 99 (52.7%) dogs had OE and 89 (47.3%) did not show any sign of the disease. Regarding the sex and neutering status in combination with the presence of OE, no significance could be identified.

### 3.2. Breeds

An association between the breeds and the presence of OE could be determined (Pearson-Chi² *p* < 0.001) with all breeds and groups, as presented in Table 1.

As some of the datasets of breeds did not include more than five pieces of data, the five most represented breeds (with OE and without) regarding the total numbers (n = 100) were used for further analysis. These breeds were Retriever (both Golden Retriever and Labrador Retriever), Rhodesian Ridgeback, Shepherd, French Bulldogs and Collies. The results still showed statistical significance (Pearson-Chi² *p* < 0.001|Fisher’s exact test *p* < 0.001), as seen in Table 2.

Consequently, a post hoc test was performed with these results. For this, the adjusted residuals in the cross tabulation with a value > 1.9 were used which, in this case, were the following breeds: Retrievers (adjusted residual = 3.3 in presented), Rhodesian Ridgeback (adjusted residual = 3.1 in non-presented) and Collie (adjusted residual = 2.2 in non-presented).

After a Bonferroni correction (new α = 0.002) followed by another Chi² test, the statistical significance was determined for the Rhodesian Ridgeback (new *p* = 0.002198) and Retrievers (0.000866).

### 3.3. Sex and Neutering Status

Like previously stated, there were no statistically significant associations found for the OE externa presentation and gender (Pearson-Chi² *p* = 0.230|Fisher’s exact test *p* = 0.258|Odds ratio [OR] 1.313 Confidence interval [CI] = 0.841–2.049) nor the neutering status in our population (Pearson-Chi² *p* = 0.783|Fisher’s exact test *p* = 0.823|OR 0.94 CI = 0.606–1.458).

In the following, the study population was divided in four sub-groups combining the sex and neutering status (i.e., male-neutered, male-intact, female-neutered and female-intact) and another analysis was run. Again, no significant associations were detected.

Lastly, tests were applied to evaluate potential associations between OE and the opposite sex and neutering status (i.e., male-intact versus female-neutered, etc.). Also here, no significance was established.

### 3.4. Weight and Age

Both the Kolmogorov–Smirnov and the Shapiro–Wilk tests declined in their normal distribution in the set of weight and age (*p* < 0.001). Graphically, histograms and Q-Q-plots confirmed these results. Consequently, the Eta coefficient test was performed and showed little strength of association in both cases (Eta age = 0.084|Eta age = 0.074).

**Figure 3:**
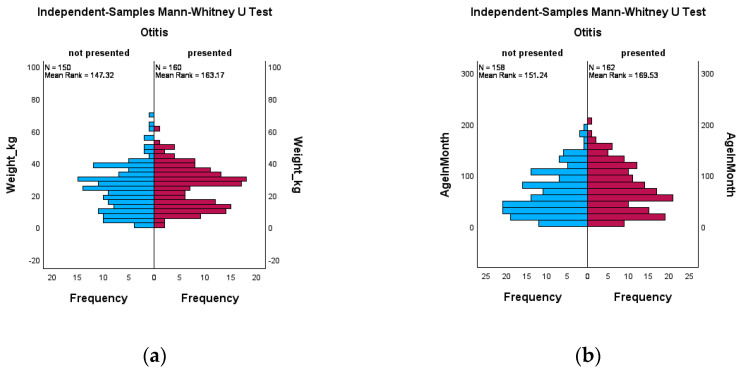
Graphical displays of data related to weight (**a**) and age (**b**), ranked according to Mann–Whitney-U testing and divided by the presence of OE. The total amount of cases in both diagrams are less than 321 as in 11 cases, no weight was assigned and one patient did not have the age incorporated.

Additionally, a Mann–Whitney-U Test was performed and the graphical results can be seen in Figure 3a,b. No significances were detected in either category.

In the following, the Pearson-Chi² and Fisher’s exact tests were applied to the categorization of the age and weight classes. These analyses also did not show any significance.

Lastly, the categories of puppy and young dog were compared to old dogs and geriatric dogs, where the same procedure was applied as for the groups of ‘less than normal weight’ (cachectic, underweight and slightly underweight) and ‘more than normal weight’ (obese, overweight and slightly overweight). An odds ratio test was performed on both ‘young dogs’ vs. ‘old dogs’ (OR = 1.364 CI = 0.799–2.327) and ‘less than normal weight’ vs. ‘more than normal weight’ (OR = 0.739 CI = 0.302–1.807).

### 3.5. Underlying Disease

As some of the dogs had multiple underlying diseases, the total amount of datasets was 353. According to the data, 114 (32.3%) dogs had an inflammatory, 11 (3.1%) a cryptogenic, 98 (27.8%) an allergic, 21 (5.9%) an endocrine, 25 (7.1%) an infectious and 10 (2.8%) a tumorous disease. Out of the 98 allergic cases, 32 (32.7%) had been diagnosed with food sensitivity and out of these, 21 cases (65.6%) had developed OE. The remaining subjects (74 (21.0%)) had an underlying or secondary disease which does not fit in the categories (e.g., disc herniation, vestibular syndrome, cruciate ligament tear and others). As previously stated, to prevent falsification due to the groups “inflammatory” and “other”, they were not included for further analysis.

After applying the Pearson-Chi² and Fisher’s exact test, a significance was shown (both *p* = 0.002), as shown in Table 3, which was still apparent for allergic (with an increased risk of OE) and infectious (with a decreased risk for OE) diseases after Bonferroni correction.

**Figure 4:**
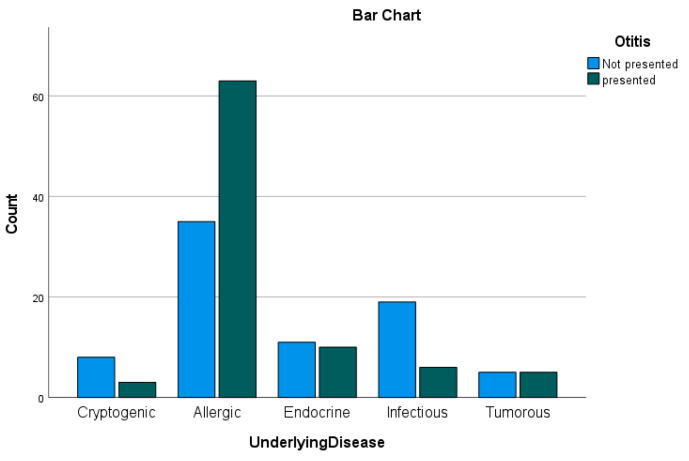
Bar chart of statistically relevant cases regarding underlying diseases. It is shown that patients with an allergic underlying disease tend to be more likely to develop OE, while less than half of the patients with infectious diseases were diagnosed with OE.

Additionally, patients with an endocrinopathy and who were overweight were compared to a group which also suffer from an endocrinopathy but were not overweight, as presented in Figure 4. It was shown that the second group has a lower risk of developing OE by odds ratio testing (OR = 0.12 CI = 0.009–1.584).

## 4. Discussion

As canine OE is a very common disease in veterinary practice, many studies exist relating to this topic [2,7,8,10,16,17,20,21,34,35,36,37]. Unlike existing ones, this study concentrated on the search of preventative factors with the aim to reduce suffering from OE in predisposed breeds.

As previously stated, patients presented multiple times were counted as one single case. This is a different approach than previous studies investigating canine otitis externa [7]. The reason was to prevent falsifying data by individual cases of severe otitis externa, treatment failure or the increased concern of pet owners.

There was no significance found between the sex or neutering status and OE, a result similarly described in other studies [15,38]. Nonetheless no clear statement should be made as yet, as some other studies suggested a predilection concerning sex [6], for example, potential contributing effects of estrogen (dry skins) and testosterone (more oily skins) are described [7], and few clinical studies regarding this specific question exist [33]. Furthermore, human medicine studies shown the potential influence of sex hormones on dermatological diseases like atopic dermatitis [39]

The distribution of canine OE among different groups of breeds showed that multiple breeds were affected. Correlating with existing studies, Retrievers showed increased odds for developing OE [38,40,41], while Collies, before post hoc testing, seemed to have a decreased risk of developing OE [7]. Furthermore, it was shown that Rhodesian Ridgebacks, even after post hoc testing, had a reduced risk. To the best of the authors’ knowledge, this is the first report of such a correlation. As evaluated in other studies, breeds with dropped ears and larger breeds (due to longer ear canals) normally tend to be more affected by OE [6]. Further studies should attempt to elucidate which specifics within the Ridgeback breed protects it from OE.

Although weight itself did not show any significance, in dogs with endocrinopathies, weight seems to influence the development of OE. After the statistical analysis, it became apparent that patients with endocrinopathy and a normal weight had a lower risk of the presence of OE than those who were overweight. Human medicine studies showed that obesity may lead to metabolic and endocrine abnormalities. Furthermore, adipose tissue is known not only for being a passive energy storage, but also as an active endocrine organ itself. Adipocytes secrete adipokines which are assumed to have proinflammatory attributes [42]. Even though the total sum of the study subjects with endocrinopathy is small (n = 14) and the range of the confidence interval is wide, to the best of the authors’ knowledge, this result is the first report of such a correlation. With that result, it would be interesting to follow endocrine-affected dogs, particularly Retrievers (with increased odds) and other breeds that tend to develop overweight on strict weight management and their relapses of OE. Should this show a significant reduction of occurrence of OE, another angle to an improved quality of life for dogs could be set, aside from the well-known benefits of normal-weight patients.

Regarding age categorization, no significance could be found. As a study in the UK has concluded, dogs under the age of one hardly suffer from OE [7]. With this in mind, it is interesting that in our study, 9 out of 19 dogs (47.4%) under the age of one did develop OE. This could be a coincidence based on the study population, but may also be caused by another factor.

The results of this study also show a significant association between the presence of OE and allergic underlying diseases, which correlates with existing data [7,15,18,19,36,43].

The literature describes flea allergy dermatitis, food hypersensitivity and environmental allergies or “atopy” as the three most common allergies in dogs [44,45,46]. Up to 90% of chronic or recurrent OE are attributed to atopic dermatitis or food allergies [47], especially against meat or dairy products [48,49,50]. Regarding pathogenesis, adverse food reactions can be divided into two groups: immunologically based (food hypersensitivity or, as a synonym, “food allergy”) and non-immunologically based (food intolerances like metabolic reactions, pharmacologic reactions and idiosyncratic reactions or as a result of toxic reactions) [48,49,51]. To understand the effect of food hypersensitivity on OE, the pathogenesis will be briefly summarized: Due to oral intake, the wall of the digestive tract is heavily exposed to the environment and, therefore, has to differentiate between nutrients and potentially harmful substances. This function is carried out by the “Gut Associated Lymphoid Tissue” (GALT) which regulates the tolerance and exclusion of antigens by mechanisms with the help of the mucosal barrier and the regulation of the immune response from antigens reaching the mucosa [50]. Abnormalities in these functions result in excessive immune responses to antigens of nutrients [50]. These responses lead to inflammation in various areas of the body unrelated to the GIT, including the skin and ear canals. Therefore, a complete medical workup is necessary to evaluate the specific hypersensitivity. The results of our study support the previous findings as a majority of the patients with food hypersensitivity developed OE. The management of OE should include feeding instructions to avoid food resulting in hypersensitivity reactions.

Studies have shown that up to 55% of dogs with atopic dermatitis seems to develop OE concurrently [45,47,52]. This disease is defined as an inflammatory and pruritic allergic skin disease often associated with the production of IgE against environmental allergens. The exact pathogenesis is unknown, but different theories including genetic predisposition, immunologic alterations and skin barrier defects are described. In the dog, predominantly, skin lesions including OE are induced by atopic dermatitis [45,53]. Besides topical treatment, allergen immunotherapy should additionally be considered to address environmental allergies [54].

Furthermore, it was shown that dogs with an infectious disease did not tend to develop OE. While this can be explained by the small number of subjects with underlying diseases (n = 25), another reason regarding ectoparasites may be focal-concentrated inflammation areas with no extent to the ear canal. But even subjects with systemic infections with multifocal dermatitis (e.g., leishmaniasis) seem to hardly develop OE. It is unknown why some of these infectious agents, even though they can cause skin diseases on several areas of the body, do not cause inflammation in the ear canals. An otic form of demodicosis is described in single cases [55]. Furthermore, *Demodex canis* has been found as a rare cause of ceruminous otitis in prior studies, supporting our study where none of the patients diagnosed with demodicosis had developed signs of OE [56]. Further studies with more study subjects and involving a microbial analysis may evaluate these findings.

## 5. Conclusions

In summary, this study showed, for the first time, that Rhodesian Ridgebacks and Collies seem to have a decreased risk for developing OE, a fact that may be interesting for potential breeders when reflecting and selecting pairs for the next breed. Furthermore, our results allude that weight has an influence on the development of OE, even if only, statistically, in combination with endocrinopathies. Nonetheless, it is well-known that obesity elicits multiple pathological mechanisms. Thus, due to well-restricted weight management by the owner, not only the quality of life, but also several aspects of the general health of a dog can be improved markedly.

All in all, this study showed that there are factors which may benefit the reduction of OE development. This is an approach which should be continued in future studies, to further evaluate prophylactic measures to improve the quality of life of our companions.

## 6. Limitations

The data collection ‘only’ ran for 2 years as the dermatology service was newly established in 2019 and older data might have included less detailed clinical evaluations. A certain bias was feared to occur when different clinicians were making the diagnostic approach. This is a common problem of retrospective studies. However, our study population is, despite the limited years, quite strong in numbers. Another limitation is the definite case bias of the referral population to the dermatology service, although the hospital saw first-opinion cases out-of-hours.

The different age-groups were used for the whole subject population without considering the life span. Bigger breeds tend to have a shorter life span than smaller breeds do.

In the case of the weight categories, the limitation was set within the system itself. To use the kilograms alone may not be enough to determine if a dog is over- or underweight. This could be specified by using a weight and height ratio or, even better, the body condition score. Both must be implemented in the clinical examination of the subjects as they guarantee a more detailed analysis while remaining an objective criterion.

As the Bonferroni correction was used for the post hoc tests, the risk of a type I error decreases but simultaneously, the risk for a type II error increases, meaning that potentially significant datasets may not be evaluated as such.

In summary, this study showed, for the first time, a statistically significantly reduced presence of OE in the Rhodesian Ridgebacks. In addition, there were increased odds of developing OE in overweight patients suffering from endocrinopathies, which should help further substantiate the importance of weight control in canine patients.

## Figures and Tables

**Table 1:** Chi-Square Test of all breeds showed significance (*p* < 0.001) regarding canine OE. (* df = degrees of freedom).

	Value	df *	Asymptotic Significance (2-Sided)
Pearson Chi-Square	45.022	19	<0.001
N of Valid Cases	321		

**Table 2:** Chi-Square Test of selected breeds which still showed significance (*p* < 0.001).

	Value	df	Asymptotic Significance (2-Sided)	Exact Sig. (2-Sided)
Pearson Chi-Square	20.483	4	<0.001	<0.001
Fisher–Freeman–Halton Exact Test	20.295			<0.001
N of Valid Cases	100			

**Table 3:** Chi-Square Test of underlying diseases shows a significant correlation between those and the presence of OE.

	Value	df	Asymptotic Significance (2-Sided)	Exact Sig. (2-Sided)
Pearson Chi-Square	16.639	4	0.002	0.002
Fisher–Freeman–Halton Exact Test	16.679			0.002
N of Valid Cases	165			

## Data Availability

The original data presented in the study are openly available on FigShare at https://doi.org/10.6084/m9.figshare.26332423.v1.
